# Structure, Dynamics, and Neural Codes in a Bat Social Network

**DOI:** 10.1111/nyas.70250

**Published:** 2026-04-07

**Authors:** Saikat Ray, Liora Las, Nachum Ulanovsky

**Affiliations:** ^1^ Department of Brain Sciences Weizmann Institute of Science Rehovot Israel

**Keywords:** bats, dominance hierarchy, neural coding, social affiliation, social neuroscience

## Abstract

Group formation and maintenance are critical for the survival of social organisms. We investigated a colony of highly social, wild Egyptian fruit bats in a laboratory‐based cave to comprehensively characterize how their social networks evolve and stabilize over weeks and months. Using state‐of‐the‐art tracking methods and videography, we documented the identities, locations, and social interactions between individual bats. We characterized the structure of social networks based on proximity‐based social affiliation and rate of social interactions—and found that the network structure evolved dynamically over a few days after the formation or alteration of the group, and subsequently stabilized. Social dominance relationships initially evolved and then remained stable over several months and were reflected in several aspects of the bats’ natural behavior, such as the monopolization of food resources and sleeping arrangements. We also conducted wireless single‐unit neural recordings in this freely behaving social colony and investigated hippocampal CA1 neurons. A subset of neurons encoded the relative (egocentric) location of other individuals and tracked the directions and distances to them. These egocentric neurons encoded more strongly high‐hierarchy bats. Overall, after an initial dynamic period of group formation, the bats established a highly structured and stable social network, which was reflected in their neural codes.

## Graphical abstract

We studied group formation in highly social Egyptian fruit bats housed in a laboratory cave. Using advanced tracking, videography, and wireless single‐unit neural recordings, we showed that social networks evolved dynamically and then stabilized, with social hierarchy shaping behaviors such as food access and sleeping arrangements. Hippocampal CA1 neurons encoded the relative locations of other bats, with stronger representations of high‐ranking individuals—linking stable social structure to neural coding.

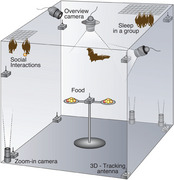

## 1 Introduction

A large number of species across taxa live in social groups, a characteristic that profoundly influences their survival and evolutionary success [1]. Social networks define key aspects of these animals’ lives, underpinning behaviors such as communication and cooperation, which produce complex group dynamics [2]. These dynamics enable essential activities like foraging [3], mating, and cooperation [4], often providing critical adaptive advantages to certain groups over others [5]. This raises a fundamental question: how do these social groups arise, evolve, and stabilize? Despite its importance, addressing this question is inherently challenging, particularly in the wild, where comprehensive monitoring and characterization of all group members and their interactions pose significant technical and logistical hurdles [6].

Among the social species, bats stand out due to their diverse and intricate social structures [7, 8]. Bats live in groups ranging from a few dozen to millions of individuals, exhibiting a variety of social networks that include kin‐based associations, cooperative alliances, and transient interactions within large roosting colonies [8, 9]. Egyptian fruit bats (*Rousettus aegyptiacus*), in particular, are a highly social species, predominantly found in the Middle East and Africa. These bats engage in an array of remarkable behaviors, including long‐distance navigation, social communication, and affiliative interactions such as allogrooming [9−13]. However, their nocturnal lifestyle, high mobility, and the size of their colonies present a formidable challenge to studying their social networks in the wild—hindering our understanding of how wild bats form and maintain such networks.

Here, we set out to address these gaps in bats—with the broader aim of shedding light on how social networks emerge and stabilize in wild animals more generally. To this end, we captured Egyptian fruit bats from the wild and housed them in a laboratory‐based cave [13]. This setup allowed us to observe and record the behavior of all individuals in a systematic manner over several months. We utilized this to gain insights into how their social networks evolved and stabilized, and we explored how hierarchies were established and maintained within the group—shedding light on the mechanisms underlying social organization in animals.

In addition to behavioral observations, we also performed wireless neural recordings from these freely flying and interacting groups of bats to investigate how their brains represented various facets of their social networks. This combined behavioral and neurobiological approach provides a comprehensive framework for understanding the dynamics of social networks in a highly social species like Egyptian fruit bats, allowing us to explore how complex social networks arise and are represented in the brain.

## 2 Materials and Methods

### 2.1 Subjects and Behavioral Setup

Fourteen adult Egyptian fruit bats, eight males and six females, were included in this study as subjects. All bats were wild‐born and were captured outdoors as adults at two different sites located 30 km apart. Electrophysiological recordings were performed on five of these bats (three males and two females). All the male bats were vasectomized to prevent pregnancies, a procedure which is well‐known not to change behavior or hormonal status in several mammalian species [14−17]. All experimental procedures were approved by the Institutional Animal Care and Use Committee of the Weizmann Institute of Science.

Bats were housed in mixed‐sex groups of 5−10 individuals. Bats 3, O, 1, 6, 7, and 2 were female, and bats X, T, A1, C, S, U, A2, and P were male. The group composition was generally stable over weeks to months and changed only to replace the recorded bats (or rarely to take out a bat for veterinary treatment). We studied five groups of bats, which had relatively equal proportions of males and females. Group 1 contained the bats: O, 1, 2, 3, C, T, U, X (U was the neurally recorded bat; the group was maintained for 6 weeks); group 2 contained the bats: O, 2, 3, C, 7, P, T, A1, X (7 and A1 were the recorded bats; 3 weeks); group 3 contained the bats: O, 2, 7, P, T (7 was the recorded bat; 8 weeks); group 4 contained the bats: O, 2, P, A2, X (A2 was the recorded bat; 7 weeks); and group 5 contained the bats: O, 2, S, 6, C, T (6 was the recorded bat, S was present for only the first few days; 10 weeks). The bat groups likely contained mostly nonkin bats, as they were captured from different wild colonies, and across a period spanning 5 years.

**FIGURE 1:**
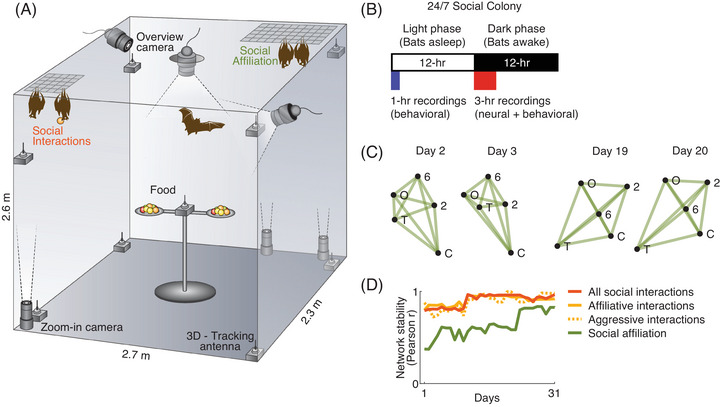
Social dynamics: the social network stabilized after a few days in a colony of freely behaving bats. (A) Social groups of 5−10 male and female wild fruit bats lived 24/7 in a room simulating a bat cave. Their social interactions and activity on nets were observed using video cameras, and they were additionally tracked in 3D throughout the room by a radiofrequency‐based 3D tracking system. (B) Schematic illustration of the daily timeline in the colony. Neural and behavioral recordings were performed for ∼3 h at the start of the bats’ awake phase (dark phase), and in addition, we performed behavioral recordings of the bat's position at the beginning of the bats’ sleep phase (light phase). (C) Example of a social network for two pairs of days: two at the beginning of the social network (days 2, 3) and two at a later stage (days 19, 20), in which this group of five bats were together. The network was constructed based on the physical distances between all pairs of bats, which is a proxy for social‐affiliation and constitutes our social affiliation index. Nodes of the graph correspond to individual bats (indicated by letters or numbers). (D) Average social network stability over a month, quantified by the correlation (Pearson *r*) between the vector of graph centralities for each recording‐day and the vector of median graph centralities of that social network across all days (*n* = 5 different networks [groups] for the five recorded bats). Network stability was computed here either based on distances between all pairs of bats (social affiliation, green), on the rate of all the social interactions with the recorded bat (social interactions, red), or on the rate of affiliative interactions or aggressive interactions with the recorded bat (solid and dashed yellow lines, respectively). Panels A and B were adapted with permission from Ref. [13].

During the study, the bats were housed in a medium‐sized room (2.7 × 2.3 × 2.6 m: Figure 1A) under controlled conditions (day‐light cycle: 12 h light/12 h dark; temperature 22±2°C). The room featured two asymmetrically sized landing nets in opposite corners (sizes: 80 × 40 cm and 40 × 40 cm), which provided convenient locations for social interactions and huddling. The bats’ diet consisted of mixed fruits, predominantly bananas, and there was no additional water source, as the bats’ water requirement was fulfilled by the water content in the fruits. Food was available *ad libitum* on two central plates, refreshed daily before recording sessions. During the dark phase, when the bats were active, the light levels were low (1 lux); and during the light phase, when the bats were asleep, a relatively bright light was present (220 lux) (Figure 1B). All experiments were conducted remotely, with no human presence in the room during the behavioral or neural recordings.

Two complementary tracking systems were employed to localize and identify the bats. The first system used lightweight ultra‐wideband radiofrequency tags placed on the bats’ heads. These tags communicated with an array of nine antennas positioned throughout the room to estimate the 3D positions of bats with 10 cm accuracy at a 25 Hz sampling rate. Each bat had a unique ID, and localization data were synchronized with neural recordings to submillisecond precision. The second system utilized six synchronized video cameras for high‐resolution tracking (1‐mm accuracy) in specific areas, primarily on the landing nets. The cameras captured 2D barcodes on the bats to record their identities, positions, and head directions. These dual systems allowed comprehensive tracking of bat behavior across the room and on the nets, with synchronized data enabling detailed analysis of spatial and social dynamics. A more comprehensive description of these systems can be found in Ref. [13].

The neural and behavioral data from the dark phase and the hierarchy test included in this study (see below) were published in Refs. [13] and [18]. Data from the light phase were not published to date.

Finally, we wish to emphasize that the bats were free to move and behave at will. There was no behavioral task imposed by the experimenters. Thus, the spatial and social behaviors reported in this study were all internally driven and fully natural.

### 2.2 Social Network Analysis

To determine the social network in each experimental session during the active phase, we used the behavioral data recorded while the bats were on the net. We assessed the stability of the social network of the bats via two types of indexes of social affiliation measured while they were on the net [13]: (1) using an affiliation index, which was based on the median distances between all possible pairs of bats during each experimental session; and (2) using the rate of social interactions—either (i) only affiliative social interaction; (ii) only aggressive social interactions; or (iii) all the social interactions involving the recorded bat and its conspecifics in each session. The social interactions were annotated based on observing the video recordings by multiple human observers (annotators) who were blind to the experimental hypotheses. The observers demarcated the type of social interaction—which could be affiliative, aggressive, joining a group, or leaving a group. They also marked the onset times of the social interactions. We determined interobserver reliability by having all the observers annotate independently the same subset of social interactions—where the observers had to both identify the onset of the social interaction and the type of social interaction. To assess agreement between observers, both the type of interaction and the timing of the interaction had to match, while a disagreement could happen due to either a mismatched interaction type or nonalignment of the interaction onset. We found that the observers showed an average agreement rate of 71%, and the timing of the matched interactions was on average within 170 ms of each other.

To determine social network stability (Figure 1C,D), we computed for each experimental session the closeness network centrality—a standard metric for quantifying the structure of graphs [13], and then calculated the Pearson correlation *r* of this daily set of values to the median graph centrality for all the days for each group of bats. The nodes of the graph represented individual bats, and the distances between the nodes in the network were taken as one of the two measures mentioned above (median distance between bats, and rate of social interactions)—thus generating two independent metrics of social network stability and dynamics. The dynamics of the social networks of this bat species were not reported to date.

### 2.3 Social Hierarchy Test

**FIGURE 2:**
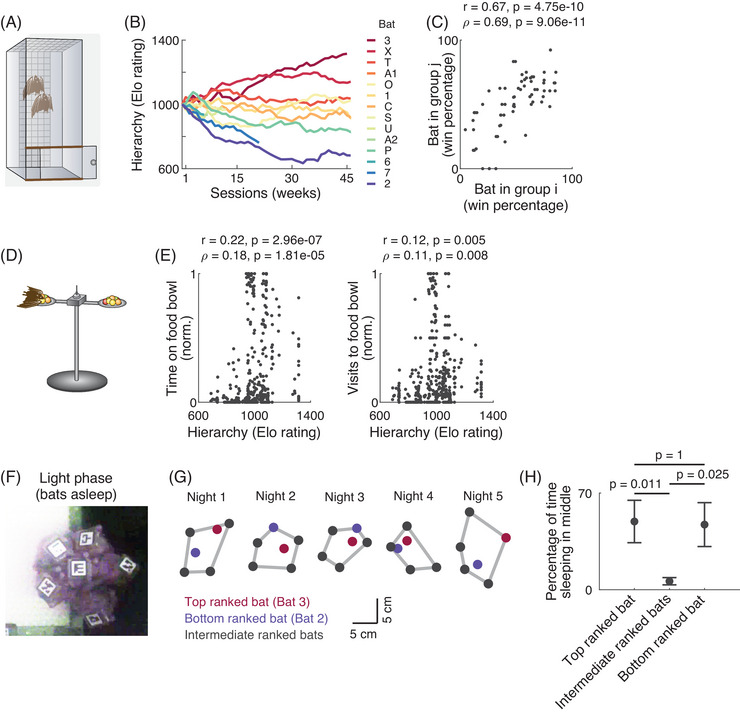
Social hierarchy: bats exhibited a stable hierarchy over months, and their hierarchy played a role in multiple behaviors. (A) A schematic illustrating the setup used for determining the dominance hierarchy within pairs of bats. Two bats were placed at the bottom of a vertical box and were allowed to climb simultaneously up to the top of the box by using a ladder placed on the box's wall. The first bat to reach the top was deemed the higher in hierarchy. All possible pairs were tested and ranked using Elo ratings. (B) Social hierarchy (Elo rating) of individual bats over time (weeks). Each curve corresponds to an individual bat (*n* = 14 bats that participated in our five groups). (C) Consistency of social hierarchy across groups. Shown is a scatter of the winning percentage of one bat against another bat in the social hierarchy test shown in A, plotted for every pair of bats (dots)—comparing performance when the pair was part of group *i* versus when the same pair was part of group *j*. The high correlation shows that the relative dominance of the bats was consistent across different overall group compositions (Pearson *r* = 0.67, *p* = 4.75×10^−10^; Spearman *ρ* = 0.69, *p* = 9.06×10^−11^; *n* = 67 bat‐pairs across the five groups). (D) A schematic illustrating a bat occupying the food bowl. (E) Relation between social hierarchy and food monopolization. Left, a scatter showing the Elo rating of each bat against the time it spent on the food bowl per session, normalized by the total time spent by all the bats on the food bowl in that session. The hierarchy and the time spent on the food bowl were significantly correlated (Pearson *r* = 0.22, *p* = 2.96×10^−7^; Spearman *ρ* = 0.18, *p* = 1.81×10^−5^; *n* = 538 sessions × bats [dots]). Right, a scatter showing the Elo rating of each bat against the number of visits to the food bowl per session, normalized by the total number of visits by all bats to the food bowl in that session. The hierarchy and the number of visits to the food bowl were significantly correlated (Pearson *r* = 0.12, *p* = 0.005; Spearman *ρ* = 0.11, *p* = 0.008; *n* = 538 sessions × bats). (F) An image from the light phase illustrating how the bats clustered while sleeping in a group. (G) The median position of the bats during the ∼1‐h sleep recording over five consecutive nights, showing the position of the top ranked bat in the hierarchy (bat 3, red), the bottom ranked bat in the hierarchy (bat 2, blue) and the intermediate‐ranked bats (dark‐gray dots). The gray lines denote the convex hull of the sleeping‐cluster of the bats. (H) The percentage of time spent in the middle of the group by the top‐ranked bat, intermediate‐ranked bats, or bottom‐ranked bat (Wilcoxon rank‐sum tests [*p*‐values with Bonferroni correction for two comparisons each], *p* = 0.011 [top vs. intermediate], *p* = 0.025 [bottom vs. intermediate], *p* = 1 [top vs. bottom]; *n* = 5 nights; Error bars = mean ± SEM). Note that during the sessions analyzed in panels F−H, none of the bats were implanted with a microdrive for brain recordings. Panels A, B, and D were adapted with permission from Ref. [13].

To investigate social dominance relationships within the bat colony, we designed a pairwise vertical climbing test. This test was based on the antipredator and roosting ecology observed generally in other bat species [19, 20], and utilized bats’ natural instinct to move upward and perch at higher locations to avoid predators. The setup consisted of a vertical box (30 × 30 × 100 cm) with a narrow ladder (Figure 2A). Two bats were placed at the bottom of this apparatus, and a flap was removed to start the test. Bats competed to climb the ladder, creating a “race to the top.” Weekly pairwise tests were conducted over a year, and Elo ratings, a standard index of social dominance, were calculated from the results [13] (Figure 2B,E). However, we note that roosting height has not been investigated as a dominance marker in Egyptian fruit bats in the wild, and here we evaluate its ethological relevance by comparing it to other dominance‐related behaviors, such as food resource monopolization (Figure 2D,E). Both the top‐ranked and bottom‐ranked bats (bats 3 and 2) were females, suggesting that sex and hierarchy were unrelated (see further on this below).

### 2.4 Sleep Cluster Analysis

To determine if, during sleep, specific bats were located inside or on the periphery of the cluster of sleeping bats (Figure 2F‐H), we first determined the median locations of the bats on the net during the light phase, when they were largely asleep, by tracking the 2D barcodes on each of the bats. We next determined the largest cluster of bats at each moment, and then computed the convex hull of this cluster; this convex hull was then used to assess if each individual bat was part of the periphery of the sleep‐cluster (part of the convex hull), or was located at the center of the sleep‐cluster (inside the convex hull). We note that the bats from whom we performed neural recordings were not included in these analyzed sessions, and thus, all the bats had identical implants on their heads (Figure 2F). The social‐spatial sleep arrangements of this bat species were not reported to date.

### 2.5 Neural Recording

To perform neural recordings, five bats were implanted with 16‐tetrode microdrives above the dorsal hippocampus, using previously established methods [13, 21]. Tetrodes, constructed from platinum‐iridium wires, were gold‐plated to reduce impedance, and were positioned in the dorsal CA1 pyramidal layer of the hippocampus. Neural signals were recorded wirelessly with a 64‐channel neural logger, amplified, filtered (600−7000 Hz for spikes), and sampled at 32 kHz per channel [13]. Spike sorting was performed on the basis of the relative amplitudes of recorded spikes on different channels of each tetrode, using Plexon Offline Sorter, and well‐isolated neuronal clusters were manually selected. In total, we recorded 489 well‐isolated CA1 neurons from five bats. Of the 489 recorded neurons, we analyzed 394 neurons that had ≥ 10 min and ≥100 spikes while the recorded bat was located on the large net together with ≥ 3 other conspecific bats.

### 2.6 Sociospatial Responses of Hippocampal Neurons

**FIGURE 3:**
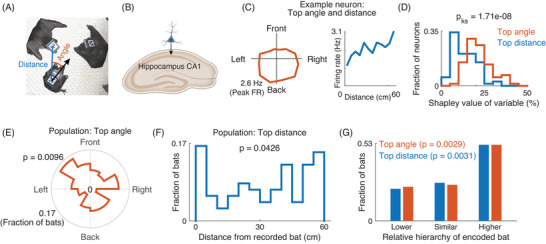
Neural coding: hippocampal CA1 egocentric neurons encoded bats higher in the hierarchy, as well as bats located in front of them. (A) Schematic illustration of the distance (blue) and direction (red) between two bats, with the distance and direction of the other bat determined from the perspective of the bat from whose hippocampus we performed neural recordings. (B) A schematic illustrating the hippocampus CA1 region. We recorded neural activity from dorsal CA1 neurons using wireless single‐unit electrophysiology. (C) An example egocentric neuron from the CA1 region of the hippocampus, showing the firing‐rate maps of the same neuron for the angle (direction) to one bat (left) and the distance to another bat (right). The two displayed bats had the highest Shapley values (explained variance) for direction (top encoded angle) and distance (top encoded distance), which were simultaneously encoded by this neuron. (D) Distributions of Shapley value (explained variance) of the top encoded angle (the other bat with highest explained‐variance for angle: red) and the top encoded distance (the other bat with highest explained‐variance for distance: blue), for all the recorded egocentric neurons. In the relatively small nets, the directions to other bats (angle) were more prominently encoded (higher Shapley value) than the distances (Kolmogorov−Smirnov test, *p* = 1.71×10^−8^; *n* = 73 neurons). (E) The distribution of preferred angles to the top‐ranking other bats (bats with highest Shapley values [highest explained variance]; preferred angle denotes the peak of the directional tuning‐curve). This distribution was significantly different from a uniform distribution (Kuiper's test, *p* = 0.0096; *n* = 73 neurons). (F) The distribution of preferred distances to the top‐ranking other bats (preferred distance denotes the peak of the distance tuning‐curve). This distribution was significantly different from a uniform distribution (χ^2^ test, *p* = 0.0426; *n* = 73 neurons). (G) Bar graphs showing whether the bat with the top encoded angle and top encoded distance was lower in hierarchy (∆Elo rating < −50), similar in hierarchy (−50 ≤ ∆Elo rating ≤ 50), or higher in hierarchy (∆Elo rating > 50) than the recorded bat. The distributions for both the bats with the top encoded angles and the bats with top encoded distances were different from a uniform distribution (χ^2^ tests: *p* = 0.0029 [top angle], *p* = 0.0031 [top distance]; *n* = 73 neurons). Panels A−C were adapted with permission from Ref. [13].

We used a generalized additive model to predict neural activity based on behavioral variables, such as positions, directions, and distances between the recorded bat and the other bats that were present on the net [13] (Figure 3). To distinguish between neurons that encoded absolute locations (allocentric) and relative locations (egocentric) of the other bats, we performed model selection using the deviance information criterion (DIC), and the model with the lower DIC was selected [13] (with a minimum |∆DIC| > 10). Overall, we found that 172 of the 394 neurons could be classified as significantly allocentric or egocentric; 73 of these 172 neurons were egocentric and represented the location of each of the other bats in relative space (i.e., the egocentric directions and distances to them). These 73 egocentric neurons were analyzed in this study.

To determine which conspecific bats affected most strongly the neuronal firing, and whether the directions or distances toward these conspecifics were the dominant factors in shaping the neuronal activity, we computed the Shapley values—a standard metric that provides the optimal estimation of how much a variable contributes to a model [13] (explained variance). The Shapley value was determined by running all possible model combinations for the variables (*2^n^* combinations for *n* variables) and determining how much each of these models explained the neural firing.

## 3 Results

We created a laboratory‐based cave to house mixed‐sex colonies of wild‐caught Egyptian fruit bats, which lived there 24/7 (Figure 1A). Each colony had an approximately equal ratio of male‐to‐female bats, and contained between 5 and 10 individual bats, with some individuals being present across multiple colonies. The group composition remained stable over extended periods of time (weeks to months) after the colony was established, and we tracked overall five colonies of bats. In this continually housed colony, we performed behavioral and neural recordings for ∼3 h per day at the start of the dark phase when the bats are most active (Figure 1B, *n* = 99 sessions). We also performed behavioral tracking for ∼1 h during the start of the light phase, when the bats were asleep (*n* = 5 sessions). All the bats were tracked individually using two systems: (i) a radiofrequency tracking system—which tracked all the bats in three dimensions at all times at 10‐cm resolution during the 3 h session; and (ii) a video‐based 2D barcode tracking system—which tracked all the bats at mm‐resolution using cameras while they were on the two landing nets at the top corners of the room. This tracking was performed in both dark‐phase and light‐phase sessions.

We first investigated how the social network of the bats evolved dynamically over days, from the first day of the group‐establishment. To this end, we evaluated the structure of the social network using two types of indexes: (1) *social affiliation*—based on the pairwise distances between all possible pairs of bats, that is, their proximity to each other during a day (Figure 1C); (2) *rate of social interactions*—with three variants of this index: (i) rate of all social interactions; (ii) only affiliative social interactions; or (iii) only aggressive social interactions between the recorded bat and all other bats. Using these indexes, we then determined how the structure of the social network dynamically evolves over time. For each index, we compared the structure of the social network on each day to the structure of the median social network across all the days, separately for each colony. This was done by computing the Pearson correlation *r* between the graph centrality of the social network for each day to the median graph centrality for that colony across all the days. We then determined the average of these correlations for the different bat colonies (Figure 1D, *n* = 5 bat colonies). We found that based on all of these indices—the social network gradually stabilized over several days after the colony commenced (example: Figure 1C, population: Figure 1D).

Social animals often display hierarchical structures. To investigate the social hierarchy of bats, we leveraged the natural instinct of bats to perch at higher locations as a strategy to avoid predators [10, 20] (see Methods). Based on this behavior, we designed a social hierarchy test for the bats, which we conducted once a week separately from the usual social colony setup. The hierarchy testing was done on pairs of bats. The setup consisted of a narrow vertical box, which was too confined for flight, but had a ladder inside that allowed the bats to climb up (Figure 2A). The test began by placing two bats at the bottom of the box, and then a flap was removed to grant them access to the ladder—resulting in a “race to the top.” We then conducted this pairwise test between all possible pairs of bats weekly over approximately 1 year. To quantify the hierarchy, we analyzed the outcomes of the pairwise tests using Elo ratings—a standard measure of social dominance hierarchy. We found that the bats exhibited a stable social hierarchy over many months (Figure 2B).

We next assessed the consistency of this social hierarchy in two ways. We first determined how consistent was the social hierarchy between two specific pairs of animals, when the same pair of bats was part of different overall group combinations, and found that the win‐loss ratios of bat pairs were highly correlated across different groups (Figure 2C; Pearson correlation: *r* = 0.67, *p* = 4.75×10^−10^; Spearman correlation: *ρ* = 0.69, *p* = 9.06×10^−11^; *n* = 67). We also determined the transitivity of their dominance hierarchy [22]. Transitivity implies that if bat A is more dominant than bat B, and bat B is more dominant than bat C, then bat A should also be more dominant than bat C. To measure transitivity, we re‐evaluated the Elo rating, analyzing all triads of bats that could be directly compared (i.e., groups of three bats that competed against one another in the dominance hierarchy test). For each triad, we recalculated their Elo ratings based on pairwise comparisons and found that the majority of triads were transitive (84%, *n* = 113/134 triads). The remaining 16% of nontransitive triads occurred when two bats had closely matched Elo ratings. Taken together, the bats showed stable social dominance relationships.

We next investigated whether this social hierarchy was evident in other natural behaviors of the bats while they were in the colony—such as eating and sleeping. First, we investigated how the social hierarchy was manifested in how much the bats monopolized the food bowl (Figure 2D,E). We found that the Elo rating of the bats was significantly correlated with two indices of food monopolization: (i) the fraction of time that the bat occupied the food bowl (Figure 2E, left; Pearson correlation: *r* = 0.22, *p* = 2.96×10^−7^; Spearman correlation: *ρ* = 0.18, *p* = 1.81×10^−5^; *n* = 538); and (ii) the number of visits to the food bowl (Figure 2E, right; Pearson correlation: *r* = 0.12, *p* = 0.005; Spearman correlation: *ρ* = 0.11, *p* = 0.008; *n* = 538)—implying that the bats higher in social hierarchy were monopolizing the food bowl more than the lower‐ranking bats.

Second, we investigated how the social hierarchy may have affected the spatial social sleeping arrangements of the bats. Bats, like many other social animals, typically huddle to form clusters when they sleep (Figure 2F). This prompted us to investigate if the sleeping arrangement in this cluster, during the light phase, was related to their social hierarchy. To this end, we computed the median positions of the bats during the session, and determined the convex hull of the sleep‐cluster—to classify which bats lay on the periphery of the sleep‐cluster (along the edges of the convex‐hull), and who was inside the cluster (inside the convex hull). We found that on all the days in which we recorded the colony during the light phase (*n* = 5 days), the middle of the sleep‐cluster was occupied either by the bat highest in hierarchy, or by the bat lowest in hierarchy, or by both; in contrast, the intermediate‐ranked bats always occupied the periphery (Figure 2G). Specifically, on four out of five nights, the bat highest in the social hierarchy spent most of the time in the middle of the cluster, while on three out of five nights, the bat lowest in the social hierarchy spent most of the time in the middle. Overall, the top or bottom ranked bats spent a significantly greater time in the middle of the sleep‐cluster, with the intermediate‐ranked bats barely spending any time in the middle of the cluster (Figure 2H; Wilcoxon rank‐sum tests [top vs. intermediate]: *p* = 0.011, *W* = 106; [bottom vs. intermediate]: *p* = 0.025, *W* = 102; [top vs. bottom]: *p* = 1, *W* = 26; all *p*‐values were Bonferroni‐corrected for two comparisons each; *n* = 5 nights).

We next asked how the brain kept track of the social network. We specifically investigated the epochs when the bats spent time on the net during the active phase and were observing the other bats without explicit social interactions (Figure 3A). To achieve this, we conducted wireless single‐unit neural recordings from the dorsal hippocampal CA1 region (Figure 3B). We analyzed the responses of each neuron by using a generalized additive model, where we used the behavioral information—specifically, the directions and distances to each of the other bats—to determine the neural response [13]. We also evaluated the Shapley values to determine how much each of these behavioral variables explained the neural firing. We found that some dorsal hippocampal neurons represented the egocentric locations of multiple other bats relative to themselves and encoded the directions or distances toward them (Figure 3C). These egocentric‐coding neurons accounted for 18.5% of the CA1 neurons (*n* = 73/394 neurons). Interestingly, on the relatively small nets (80 × 40 cm and 40 × 40 cm), we found that the hippocampal CA1 neurons more strongly tracked the directions toward the other bats than the distance to them (Figure 3D; Kolmogorov−Smirnov test, *p* = 1.71×10^−8^; *D*_KS 73, 73_ = 0.49; *n* = 73 neurons).

We then investigated how the bats that were most prominently represented by the neurons (i.e., the conspecific bats with the highest Shapley values for direction and distance) were spatially oriented with respect to the recorded bat. To this end, for each neuron, we first identified the conspecific bats that had the highest Shapley values for angle and distance. We then used the direction and distance tuning‐curves for these conspecifics to determine the coordinates (orientation and distance) at which the neuron responded most strongly to the conspecific bats. We found that the tuning for both the direction and distance of these other bats were not uniformly distributed (direction: Figure 3E, Kuiper's test compared to uniform distribution, *p* = 0.0096; *V* = 0.19; distance: Figure 3F, χ^2^ test compared to uniform distribution, *p* = 0.0426; χ^2^(11) = 20.21; *n* = 73 neurons). Specifically, the other bats were most prominently encoded by the neurons when they were in front of the recorded bats rather than behind them (Figure 3E, see “Front” half; Wilcoxon signed‐rank test comparing whether there are more neurons in the front hemicircle vs. the back hemicircle: *p* = 0.0466; *W* = 1665). Additionally, these neurons also tended to encode other bats that were either close or far away (Figure 3F; note the U‐shape of this distribution).

We next investigated whether these egocentric hippocampal neurons encoded any sex‐specific information—either differentiating between males and females or differentiating between conspecifics of the same sex or opposite sex as the recorded bat. We found no evidence of sex differences in the neural encoding of the conspecific bat that had the highest Shapley value—neither for coding the angle (splitting the distribution in Figure 3E by male and female bats or by same‐sex and opposite‐sex bats and comparing these distributions [Kuiper's test]: male vs. female bats, *p* = 0.89; *V* = 0.14; same‐sex vs. opposite‐sex bats, *p* = 0.07; *V* = 0.29), nor for coding the distance (similar splits for distance in Figure 3F [Kolmogorov−Smirnov test]: male vs. female bats, *p* = 1.0; *D*_KS 32, 41_ = 0.09; same‐sex vs. opposite‐sex bats, *p* = 0.70; *D*_KS 40, 33_ = 0.16). In addition, we found that, behaviorally, sex and social hierarchy were not significantly related (Wilcoxon rank‐sum test between hierarchy of male and female bats, *p* = 0.09; *W* = 10,268), and there was no evidence for sex‐differences in the stability of social hierarchy (Wilcoxon rank‐sum test between standard deviations of dominance ranks of individual bats across sessions—comparing male vs. female bats, *p* = 0.49; *W* = 54).

Finally, we asked which bats were most prominently encoded by the egocentric hippocampal CA1 neurons, in relation to their dominance hierarchy. We found that, both for the directions and distances toward other bats, the most strongly encoded bats were those who were ranked higher in hierarchy than the recorded bat (Figure 3G, “higher”; χ^2^ tests compared to uniform distribution; [angles]: *p* = 0.0029, χ^2^(2) = 11.70; [distances]: *p* = 0.0031, χ^2^(2) = 11.53; *n* = 73 neurons).

## 4 Discussion

In this study, we explored the social network structure, dynamics, and hierarchy in laboratory‐housed colonies of wild Egyptian fruit bats—and also examined the neural encoding of their social relationships. This was a follow‐up of our previous study on social coding in groups of these bats [13]. By observing wild‐caught bats in a laboratory‐based cave, we were able to systematically and comprehensively track all the individuals that formed part of the social network. These social networks evolved dynamically during the initial days of colony formation and eventually stabilized over time (Figure 1C,D)—underscoring the adaptability of social animals in forming cohesive group structures when encountering new members and environments. Further, we established a social dominance test [13] that revealed a clear and stable social dominance relationship among the bats over long periods (Figure 2B,C). This social hierarchy was not related to the sex of the animal, with both the top‐ranked and bottom‐ranked bats in the colony being females (bat 3 and bat 2, respectively).

We revealed here a connection between the bats’ social hierarchy (dominance relationship) and several natural behaviors of the bats—such as food monopolization (Figure 2D,E) and sleeping arrangements (Figure 2F‐H). Specifically, we found a correlation between the social hierarchy and monopolizing the access to food (Figure 2D,E). Higher‐ranked bats spent more time at the food bowl and went there more frequently, reflecting a direct translation of social rank into resource access. Such dominance hierarchies have been observed in other social animals, such as primates [23]; however, they have not been well characterized in bats.

Sleeping arrangements have been previously associated with providing adaptive benefit to social species [24]—with central sleeping locations associated with better sleep, better thermoregulation, and higher safety from predators [25]. Thus, central sleeping spots are often associated with a high dominance hierarchy. In accordance with this, we also found that in bats, the top‐ranked bat often occupied central locations in sleeping clusters (Figure 2G, red dot). However, in highly social species, not only does the dominant animal benefit from protection, but also the weakest animals are often protected [26]. Interestingly, we found that the lowest‐ranked bat was also often included in the central sleeping location in the sleep cluster (Figure 2G, blue dot). Overall, only the top‐ranked or the bottom‐ranked bats slept in the middle of the cluster (Figure 2G,H). Thus, the sleeping arrangements of the bats revealed further nuances of social organization in bats, with both the highest and lowest ranked bats occupying central positions in sleep clusters. However, we note that social dominance in animals is multifaceted and context‐dependent [27, 28]—and while predator‐avoidance, roosting ecology, resource access, and sleep locations are a few natural facets where animals display social hierarchies, future studies would be required to investigate if such hierarchies also translate to other contexts in Egyptian fruit bats.

Finally, we established how hippocampal dorsal CA1 neurons encoded different individuals in the colony [13]. We characterized here a subset of neurons that represented the egocentric locations of multiple other individuals relative to themselves—encoding the directions and distances to other bats. On the relatively small nets, the directions toward other bats were more strongly encoded than the distances to them (Figure 3D)—perhaps because in such small spaces, the direction to another animal is more relevant than its distance. Further, bats in the front directions were more strongly encoded than bats in the back directions (Figure 3E). These findings support the general role of hippocampal neurons in encoding information that is most relevant to the situation [13, 29]. In line with our previous findings, we found that the egocentric neurons did not encode sex, but encoded social hierarchy [13], and the neural representation was biased toward bats of higher social rank (Figure 3G)—indicating that these neurons parse specific social information. Such biases may reflect the ecological importance of tracking influential individuals in maintaining social cohesion and navigating social hierarchies.

Social networks and dominance relationships in the wild are more dynamic than in our experiments, as bats in the wild can be predated, move from one colony to another, and change locations based on breeding seasons and resource availability [10, 30−33]. Thus, our findings of social network stability in our captive colonies likely provide an upper bound on what would be found in the more dynamic conditions in the wild. Importantly, our current study accounts for the influence of every individual in the social network; it would, therefore, be interesting in future studies in the wild to monitor entire social networks, rather than subsets of the bats, as typically done in studies outdoors. Interestingly, studies in captive groups have shown that bats use information about identities [34], and that social structure changes under conditions such as sickness [35]—indicating that the neural encoding of identities [13] may be used by these bats to shape their social behaviors. Additionally, an important aspect of social networks is vocal communication, and while social communication calls in Egyptian fruit bats have been characterized [12], future studies would be required in order to assess the relationship between social calls and sex, dominance hierarchy and social affiliations of the bats, and their neural bases—both in the lab [13, 36] and in the wild [37].

Overall, these results advance our understanding of how Egyptian fruit bats form complex social networks. By bridging gaps between ethology and neuroscience, this study also highlights the intricate interplay between social behavior and brain function and highlights the importance of comprehensively characterizing animal societies [23, 38] in order to understand the neural basis of social cognition.

## Author Contributions

S.R., L.L., and N.U. conceived and designed the experiments. S.R., L.L., and N.U. set up experimental systems. S.R. conducted the experiments. S.R. analyzed the data. S.R. and N.U. wrote the manuscript, with major input from L.L. N.U. supervised the project.

## Funding

N.U. is the incumbent of the Barbara and Morris Levinson Professorial Chair in Brain Research. This study was supported by research grants to N.U. from the European Research Council (ERC‐Synergy—oxytocINspace, and ERC‐Consolidator—NATURAL_BAT_NAV), and by research grants to N.U. from the Dr. Lou Siminovitch Laboratory for Research in Neurobiology, Drescher Center for Research on Mental and Emotional Health, Zuckerman Center for Learning, Memory & Cognition, Center for Research on Perception and Action, Dorraine S. Schwartz, and Debra and Paul Sagues; by research grants to N.U. and L.L. from the National Institutes of Health (NIH R01—NS121413), CRCNS NSF‐BSF (BSF 2020806), and Israel Science Foundation (ISF 1920/18); by the André Deloro Prize for Scientific Research and the Kimmel Award for Innovative Investigation to N.U.; and by postdoctoral fellowships to S.R. from the European Molecular Biology Organization (ALTF 853–2017) and Human Frontiers Science Program (LT000365‐2018L).

## Conflicts of Interest

The authors declare no conflicts of interest.

## Data Availability

The data used in this study are archived on the servers of the Weizmann Institute of Science and are available from the corresponding author on reasonable request.
